# Modeling RNA interference in mammalian cells

**DOI:** 10.1186/1752-0509-5-19

**Published:** 2011-01-27

**Authors:** Giulia Cuccato, Athanasios Polynikis, Velia Siciliano, Mafalda Graziano, Mario di Bernardo, Diego di Bernardo

**Affiliations:** 1 Telethon Institute of Genetics and Medicine (TIGEM), Naples, Italy; 2 Department of Engineering Mathematics, University of Bristol, Bristol, UK; 3 Department of Computer and Systems Engineering, University of Naples Federico II, Naples, Italy

## Abstract

**Background:**

RNA interference (RNAi) is a regulatory cellular process that controls post-transcriptional gene silencing. During RNAi double-stranded RNA (dsRNA) induces sequence-specific degradation of homologous mRNA via the generation of smaller dsRNA oligomers of length between 21-23nt (siRNAs). siRNAs are then loaded onto the RNA-Induced Silencing multiprotein Complex (RISC), which uses the siRNA antisense strand to specifically recognize mRNA species which exhibit a complementary sequence. Once the siRNA loaded-RISC binds the target mRNA, the mRNA is cleaved and degraded, and the siRNA loaded-RISC can degrade additional mRNA molecules. Despite the widespread use of siRNAs for gene silencing, and the importance of dosage for its efficiency and to avoid off target effects, none of the numerous mathematical models proposed in literature was validated to quantitatively capture the effects of RNAi on the target mRNA degradation for different concentrations of siRNAs. Here, we address this pressing open problem performing in vitro experiments of RNAi in mammalian cells and testing and comparing different mathematical models fitting experimental data to in-silico generated data. We performed in vitro experiments in human and hamster cell lines constitutively expressing respectively EGFP protein or tTA protein, measuring both mRNA levels, by quantitative Real-Time PCR, and protein levels, by FACS analysis, for a large range of concentrations of siRNA oligomers.

**Results:**

We tested and validated four different mathematical models of RNA interference by quantitatively fitting models' parameters to best capture the in vitro experimental data. We show that a simple Hill kinetic model is the most efficient way to model RNA interference. Our experimental and modeling findings clearly show that the RNAi-mediated degradation of mRNA is subject to saturation effects.

**Conclusions:**

Our model has a simple mathematical form, amenable to analytical investigations and a small set of parameters with an intuitive physical meaning, that makes it a unique and reliable mathematical tool. The findings here presented will be a useful instrument for better understanding RNAi biology and as modelling tool in Systems and Synthetic Biology.

## Background

RNA interference (RNAi) is a well characterized regulatory mechanism in eukaryotes [1-3] as well as a powerful tool for understanding gene function, thanks to the discovery that synthetic small interfering RNA oligomers (siRNAs) can efficiently induce RNAi in mammalian cells [4,5]. RNAi has also been used extensively as a novel "biological part" to design synthetic biological circuits in synthetic biology [6,7]. Artificial gene silencing has the potential to become a major genetic-based therapeutic tool for viral infections [8,9], cancer [10] or inherited genetic disorders [11-13].

Despite its widespread experimental application, the best way to quantitatively model RNA interference is still under debate. In systems and synthetic biology, mathematical models are essential to carry out in silico investigations of biological pathways, or novel synthetic circuits. The aim of this work is to find the most appropriate quantitative mathematical model that can correctly describe the RNAi phenomenon in mammalian cells, for varying concentrations of the siRNA oligomers.

**Figure 1:**
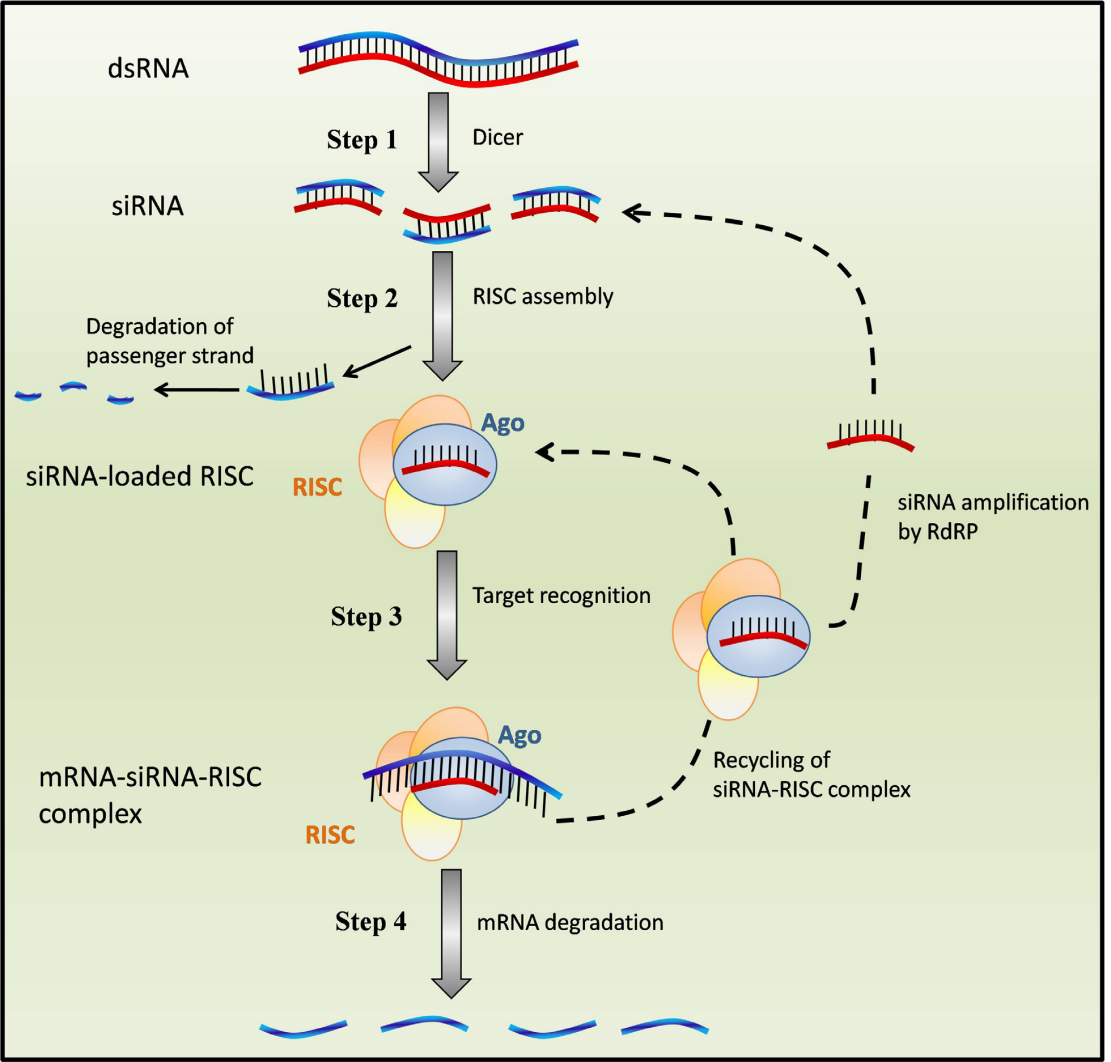
**Schematic representation of RNA interference in a mammalian cell**. Step 1: double stranded RNA (dsRNA) elicits a response in the cell mediated by the enzyme Dicer, which cleaves the dsRNA into fragments of 21-23 base pairs (siRNA). Step 2: siRNAs are loaded into a multiprotein complex called RNA Induced Silencing Complex (RISC) and one strand (the passenger strand) is discarded and degraded [14], while the guide strand remains within RISC as template in the silencing reaction. Step 3: the guide strand assembles into a functional siRNA-RISC complex, which contains the siRNA bound to the Ago protein [1]. Targets mRNAs are then recognized by Watson-Crick base pairing [1] and bound by the siRNA-RISC complex. Step 4: mRNA degradation is induced, the target mRNA is dissociated from the siRNA, and the siRNA-RISC complex is released to process further mRNA targets [15].

A schematic representation of the RNA interference mechanism is illustrated in Figure 1. In step 1, the presence of double stranded RNA (dsRNA) elicits a response in the cell mediated by the Dicer enzyme, which binds and cleaves the dsRNA into fragments of 21-23 base pairs, called small interfering RNA (siRNA). In step 2, siRNAs are loaded onto a multiprotein complex called RNA Induced Silencing Complex (RISC) and then separated into single strands of which one (the passenger strand) is discarded and degraded [14], while the guide strand remains within RISC and serves as a template in the silencing reaction. In step 3, the guide strand assembles into a functional siRNA-RISC complex, which contains the siRNA bound to the Ago protein [1]. Target mRNAs are then recognized by Watson-Crick base pairing [1] and bound by the siRNA-RISC complex. Finally, in step 4, mRNA degradation is induced, target mRNA is dissociated from the siRNA, and the siRNA-RISC complex is released to process further mRNA targets [15]. Here, we will focus on quantitatively modeling step 2 to step 4 of the RNA interference process using, as an experimental tool, synthetic siRNA oligomers.

## Results and Discussion

In order to model the effects of RNA interference on mRNA expression levels at different concentration of siRNA oligomers, we carried out in-vivo experiments of RNA interference on two mammalian cell-lines stably expressing the EGFP protein or the tTA protein, respectively.

**Figure 2:**
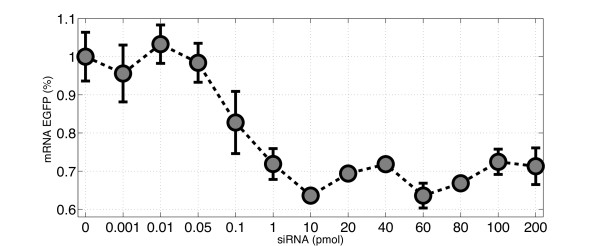
**Ratio of *EGFP *mRNA levels between cells transfected with the siRNAs specific for EGFP, and negative control cells, transfected with a non-specific siRNAs, measured 48 hours after transfection**. Errorbars represent the standard-error from three biological replicates for each point. The x-axis reports the different quantities of siRNA oligomers tested. mRNA levels were measured using real-time PCR. The error-bars have the length of one standard error.

**Figure 3:**
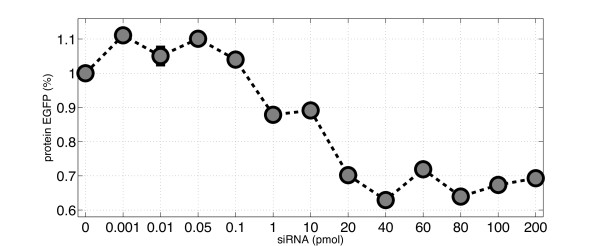
**Ratio of EGFP protein levels between cells transfected with the siRNAs specific for EGFP, and negative control cells, transfected with a non-specific siRNAs, measured 60 hours after transfection**. Error-bars represent the standard-error from three biological replicates for each point. The x-axis reports the different quantities of siRNA oligomers tested. Protein levels were measured using FACS analysis quantifying EGFP protein fluorescence. The error-bars have the length of one standard error.

In the first set of experiments (set I), Human Embryonic Kidney cells stably expressing EGFP (HEK293-EGFP cell-line), were transfected with varying quantities of synthetic siRNA oligomers directed against the *EGFP *mRNA in the range of 0 to 200 pmol. Figure 2 shows the ratio of *EGFP *mRNA levels between treated cells and negative control cells (i.e. transfected with a non-specific siRNA oligomers) measured 48 hours after transfection. Error-bars represent the standard-error from three biological replicates for each point. Similarly, in the second set of experiments (set II), EGFP protein levels were measured via FACS analysis 60 hours after transfection of HEK293-EGFP cells with synthetic siRNA oligomers directed against the *EGFP *mRNA, or with non-specific siRNA oligomers as negative controls. The ratio between EGFP protein levels in siRNA-treated cells versus negative controls, for the same siRNA quantities as in the set I experiments, is reported in Figure 3. In the third set of experiments (set III), we tested Chinese Hamster Ovary cells (CHO AA8) stably expressing the tetracycline-regulated transactivator tTA at a low level, using the same protocol used previously for HEK cells. Cells were transfected with varying quantities of synthetic siRNA oligomers against the *tTA *mRNA in the range of 0 to 200 pmol. Since the tTA is not fluorescent, no FACS measurement were performed in this cell-line. In Additional file 1 Figure 1we shows the ratio of *tTA *mRNA levels between cells treated with the silencing tTA oligomer and cells treated with the negative control (non-targeting shuffled siRNA).

### RNAi Modeling

We were interested in formulating a model that can quantitatively describe the effects of varying quantities of siRNA oligomers onto the degradation of the target mRNA species, and of its corresponding protein product. A general dynamical model describing transcription of the mRNA species, its siRNA-mediated degradation, and translation of its protein products, can be described by a system of ordinary differential equations (ODEs). Let *X_m_, X_p _*and *X_s _*be the mRNA, protein and siRNA concentrations, respectively. The evolution of their time-dependent concentrations can be described by the following ODEs:

$$\left[\text{mRNA}\right]:\;\;\frac{dX_{m}}{dt}=k_{m}-d_{m}X_{m}-\delta \left(X_{m},X_{\text{s}}\right)$$

(1) $$\left[\text{protein}\right]:\;\;\frac{dX_{p}}{dt}=k_{T}X_{m}-d_{p}X_{p},$$

The parameter *k_m_*, represents the transcription rate from the promoter that transcribes the mRNA species targeted by the siRNA oligomer; *dm *represents the basal degradation rate of the mRNA species. RNAi can be considered as a mechanism that enhances the degradation of the targeted mRNA, therefore the function *δ *(*X*_*m*_, *X_s_*) is an extra degradation term that represents the rate at which mRNAs are degraded due to RNAi. This function, *δ *(*X_m_, X_s_*), depends on both the mRNA and siRNA levels, *X_m _*and *X_s _*respectively. The parameter *k_T _*is the protein translation rate, whereas *d_p _*represents the basal protein degradation rate. At least four different models have been proposed in the literature, for the RNA interference mechanism [15-17]. Each of these models is based on the general approach described by equations (1) but each has a different functional form for *δ *(*X*_*m*_, *X_s_*). Table 1, lists all the different models studied here with a description of their corresponding parameters.

**Table 1:** The different models RNA interference models for the RNAi-induced mRNA degradation rate *δ ***(*X*_*s*_, *X*_*m*_) **and their corresponding parameters.

Model 1	
$\delta (X_{m},\;X_{s})=k_{1}X_{s}X_{m}$	*k*_1_: Rate of mRNA-siRNA* complex formation

Model 2	

$\delta (X_{m},\;X_{s})=k_{2}X_{s}^{h_{2}}X_{m}$	*k*_2_: Rate of mRNA-siRNA* complex formation *h*_2_: Number of siRNA target sites

Model 3	

$\delta (X_{m},\;X_{s})=\frac{c_{3}k_{3}h_{3}X_{s}}{c_{3}+k_{3}h_{3}X_{m}}X_{m}$	*k*_3_: Rate of mRNA-siRNA* complex formation *c*_3_: Cleavage and dissociation rate of mRNA-siRNA* h_3_: Number of siRNA target sites

Model 4	

$\delta (X_{m},\;X_{s})=d_{4}\frac{X_{s}^{h_{4}}}{\theta_{4}^{h_{4}}+X_{s}^{h_{4}}}X_{m}$	*d*_4_: Maximal degradation rate of the mRNA due to RNAi *θ*_4_: Michaelis-Menten like constant *h*_4_: Number of siRNA target sites


#### Model 1: The stoichiometric model

One possible way of modeling the effects of RNAi on the mRNA degradation is to consider a stoichiometric reaction between the siRNAs and mRNAs. Let siRNA* denote the concentration of the siRNA-RISC complex, namely the fraction of the siRNAs that are loaded into RISC complexes (step 2 of Figure 1). Then the stoichiometric reaction between the siRNA* and the mRNA (step 3 and 4 of Figure 1) can be described as follows:

(2) $$\left[{\text{siRNA}}^{*}\right]+\left[\text{mRNA}\right]\leftarrow \left[\text{mRNA}-{\text{siRNA}}^{*}\right]\to \emptyset .$$

Namely, the siRNA-loaded RISC binds to the complementary mRNA and then both are degraded. According to this model, following the law of mass action, we predict that the siRNA mediated degradation will be proportional to the product of the concentration of siRNA oligomers and the targeted mRNA species:

(3) $$\delta \left(X_{m},X_{\text{s}}\right)=k_{1}X_{m}X_{\text{s}}$$

where parameter *k*_1 _represents the proportionality constant. In this modeling approach, the siRNA-RISC complex is assumed not to be recycled, but the RISC needs to be reloaded before it can degrade another mRNA molecule (i.e. in this model the dashed line linking step 4 to step 3 in Figure 1 is not taken into account). Indeed, this model was suggested for RNA interference in prokaryotes [16,18-20]. RNAi in prokaryotes has important differences with RNAi in eukaryotes. One of these is that the interaction between the siRNA and its mRNA target is non-catalytic in nature [16,21]. This is not the case in mammalian cells. Once the mRNA is cleaved, the siRNA-RISC complex is dissociated from it, in an ATP-dependent manner [22] and it is free to process further targets [1,14,23]. Additionally, in some organisms, such as C.elegans, the primary dsRNA trigger induces synthesis of secondary siRNAs (if the target mRNA is present) through the action of RdRP enzymes, strengthening and perpetuating the silencing response [1,24].

#### Model 2: Stoichiometric model with co-operativity

This model is a straightforward extension of Model 1, which additionally takes into account the presence of multiple sites on the targeted mRNA where the siRNA-loaded RISC can bind. Model 1 can be easily extended to include co-operativity:

(4) $$\left[\text{mRNA}\right]+\underset{h_{2}}{\underset{︸}{\left[\text{siRNA*}\right]+...+\left[\text{siRNA*}\right]}}↔\text{mRNA}-h_{2}\text{siRNA}*\to \emptyset$$

As before, the rate of RNAi-driven degradation, can be easily obtained applying the law of mass action:

(5) $$\delta \left(X_{\text{s}},X_{m}\right)=k_{2}X_{\text{s}}^{h_{2}}X_{m}$$

where *k*_2 _is the proportionality constant and *h*_2 _is the number of siRNA binding sites on the targeted mRNA species. This model was suggested in [17] for modeling RNAi by miRNAs, since it is experimentally proven that multiple sites for the same miRNA can boost target mRNA repression [25]. This model however suffers from the same limitations as Model 1.

#### Model 3: Enzymatic model

A detailed model of RNAi specific for mammalian cells was proposed by Malphettes *et al *[15]. This model accounts for the catalytic nature of RNAi in mammalian cells, thus modeling step 2, step 3 and step 4 of Figure 1, including the recycling of the RISC complex (dashed line from step 4 to step 3). Specifically, it assumes that once the cleavage and degradation of the targeted mRNA is completed, the siRNA-RISC complex (siRNA*) dissociates from its mRNA target, and it is free to degrade further mRNA molecules. Additionally, this model also considers that the targeted mRNA may have a multiple number, *h*_3_, of siRNA binding sites. The siRNA-RISC complex (siRNA*) can bind to any site on the mRNA to form an intermediate mRNA-siRNA* complex, which can either accommodate further siRNA-RISC complexes on any other free binding sites, or cleave the target mRNA and dissociate from the cleavage products. The reaction of the complex formation of the target mRNA with the siRNA-RISC complex is described as follows (for details refer to [15]):

(6) $$\left[{\text{siRNA}}^{*}\right]+\left[\text{mRNA}\right]↔\left[\text{mRNA}-{\text{siRNA}}^{*}\right].$$

The model considers the following reaction between an intermediate mRNA-siRNA* _*i*-1 _complex with another siRNA-RISC complex (for all *i *∈ [1 : *h*_3_]):

(7) $$\left[{\text{siRNA}}^{*}\right]+\left[\text{mRNA}-{\text{siRNA}}_{i-1}^{*}\right]↔\left[\text{mRNA}-{\text{siRNA}}_{i}^{*}\right].$$

The generic cleavage and degradation reaction of the mRNA by any interacting siRNA-RISC complex (∀*i *∈ [1 : *h*_3_] is represented by:

(8) $$\left[{\text{siRNA}}_{i}^{*}\right]\;+\;\left[\text{mRNA}\right]\;\to \;\text{cleaved}\;\text{mRNA}+i\left[{\text{siRNA}}^{*}\right].$$

In [15], the authors show that the rate of RNAi-driven mRNA degradation, *δ *(*X_m_, X_s_*), is given by:

(9) $$\delta \left(X_{s},\;X_{m}\right)=\frac{c_{3}k_{3}h_{3}}{c_{3}+k_{3}h_{3}X_{m}}X_{s}X_{m}$$

where *k_3 _*is the rate of mRNA-siRNA* complex formation for a single siRNA target site (reaction 7) and *c*_3 _is the cleavage and dissociation rate of mRNA-siRNA* complex (reaction 8). This functional form, for a constant *X_s_*, is a classic Michaelis-Mentenu enzymatic reaction, as can be observed by simply defining *V_m _= c_3_X_s _*and $K_{m}=\frac{c_{3}}{k_{3}h_{3}}$, and rewriting Eq. (9) as:

(10) $$\delta \left(X_{s},\;X_{m}\right)=V_{m}\frac{X_{m}}{K_{m}+X_{m}}$$

This is in perfect agreement with the experimental finding on the enzymatic activity of both non-mammalian and mammalian RISC on mRNA degradation, as reported in [23,26,27], where the product of the reaction (degraded mRNA) was measured at varying the concentration of the substrate (non-degraded mRNA *X_m_*) for a *constant *amount of the RISC enzyme.

Model 4 basically assumes that only the maximal rate *V_m _*of the Michaelis-Menten will be affected when the concentration of the active RISC (proportional to *X_s_*) changes. In [23,26] the authors show experimentally that changing the concentration of RISC indeed changes the value of *V_m _*but also of *K_m_*. This is not captured by this model.

Note also that when *X_m _*≪ *K_m_*, i.e. low amounts of mRNA compared to the Michaelis-Menten constant *K_m_*, the model can be simplified to: $\delta \left(X_{s},X_{m}\right)=\frac{V_{m}}{K_{m}}X_{m}=k_{3}h_{3}X_{m}X_{s}$. This is equivalent to Model 1. On the other hand, when the mRNA concentration is very high, the model can be simplified to: *δ (X_s_, X_m_) = V_m _= c*_3_*X_s_*. Namely, the rate of RNAi-driven mRNA degradation will depend only on the amount of active enzyme, i.e. the siRNA concentration. The higher the siRNA concentration, the higher the degradation rate that can be achieved, without any saturation effect.

#### Model 4: Phenomenological model

In [17] the authors proposed a standard Hill-kinetic model to describe the post-transcriptional effects of microRNAs on the gene expression. miRNAs are processed by the cell to produce dsRNAs which then follow the typical RNAi pathway, schematically described in Figure 1[15,28,29]. By considering a Hill-type enzymatic model with an Hill coefficient *h *≥ 1, the model can be extended to account, either, for multiple binding sites of the siRNA on the same target mRNA, or, for the cooperativity of protein complexes involved in RNAi [17]. Differently from the other three model presented above, this model is a phenomenological model not derived from specific biochemical reactions:

(11) $$\delta \left(X_{s},X_{m}\right)=d_{4}\frac{X_{s}^{h_{4}}}{\theta_{4}^{h_{4}}+X_{s}^{h_{4}}}X_{m}$$

This model, despite being phenomenological has interesting properties. The kinetic parameters *d*_4 _and *θ*_4 _depend on the efficiency of siRNA binding to its sites on the target mRNA [17]: *d*_4 _represents the maximal degradation rate of the mRNA due to RNA interference; *θ*_4 _the concentration of siRNA oligomers needed to achieve half of the maximal degradation rate. The above equation implies that for *X_s _*≪ *θ*_4_, the increase in the RNAi mediated degradation is linear with $X_{s}^{h_{4}}$ (namely, it will be identical to Model 1 for *h*_4 _= 1, or to Model 2 for *h*_4 _= *h*_2_), while it saturates at higher levels of *X_s_*, reaching the maximal degradation rate *d*_4_, differently from Model 3.

### Parameter Identification

The four models were fitted to the three mRNA and protein experimental datasets (I, II and III), by searching for the parameter values for which the model-generated data best fitted the experimental data, according to a squared error measure. The results of the fitting procedure for each of the models, together with the optimized values of their parameters, are given in Table 2 for EGFP and in Additional file 1 Table A1 for tTA.

**Table 2:** Numerical fitting results of the four models for in vitro experimental data for the EGFP protein and mRNA.

		Experiment on *EGFP *mRNA levels			
	**Fit Err**.	**Pred. Err**.	**Parameters**		

**Model 1**	1.00	0.98	*k*_1 _= 1.38 × 10^-4^(pmol min)^-1^,		
**Model 2**	0.13	0.12	*k*_2 _= 5.00 × 10^-3^(pmol^*h*2 ^min)^-1^,	*h*_2 _= 0.126,	
**Model 3**	1	1	*k*_3_*h*_3 _= 1.40 × 10^-4^(pmol min)^-1^,	*c*_3_/*k*_m _= 1.33 × 10^3^a.u.,	
**Model 4**	0.04	0.05	*θ*_4 _= 0.105 pmol,	*d*_4 _= 8.1 × 10^-3^min^-1^,	*h*_4 _= 4.47

		**Experiment on EGFP protein levels**			

	**Fit Err**.	**Pred. Err**.	**Parameters**		

**Model 1**	0.97	0.81	*k*_1 _= 9.90 × 10^-5^(pmol min)^-1^,		
**Model 2**	0.46	0.39	*k*_2 _= 1.10 × 10^-3^(pmol^*h*2 ^min)^-1^,	*h*_2 _= 0.456,	
**Model 3**	1	1	*k*_3_*h*_3 _= 1.20 × 10^-4^(pmol min)^-1^,	*c*_3_/*k_m _*= 1.33 × 10^3^a.u.,	
**Model 4**	0.21	0.12	*θ*_4 _= 12.9 pmol,	*d*_4 _= 8.6 × 10^-3^min^-1^,	*h*_4 _= 4.49

The relative value of the error (Fit Error) and the relative value of the prediction error (Pred. Err.) for each model are given, together with the corresponding optimized values of its parameters. The unit of measurements are reported for the dimensional parameters. (a.u. stands for arbitrary units of concentration).

**Figure 4:**
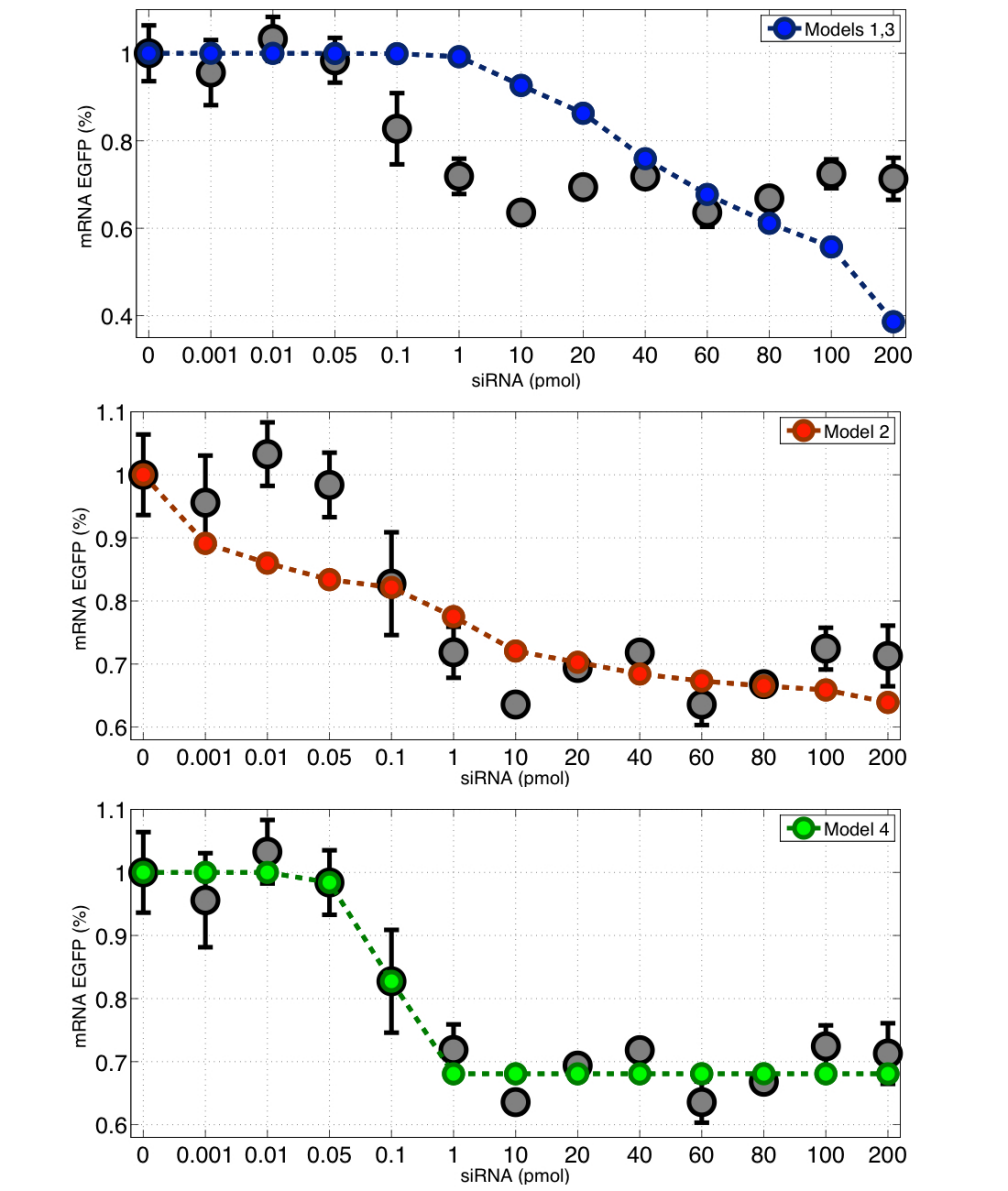
**Numerical fitting of the four models on the in vitro experimental results on mRNA *EGFP *expression levels presented on Figure 2**. The optimized parameter values and the corresponding fit error of each model are given in Table 2.

The fitting results for the mRNA levels (set I) are shown in Figure 4. Model 4 gives a significant smaller error than the other three models (see Table 2), in fact, compared to Models 1 and 3, the error of Model 4 is two orders of magnitude smaller. Model 2, gives a better fitting than Models 1 and 3, however it is worse than Model 4. The optimized value for the parameter *d*_4 _in Model 4, is *d*_4 _= 0.0081 min^-1^, indicating that the strength of siRNA mediated mRNA degradation is comparable to the strength of basal mRNA degradation (since its value is in the same order of magnitude as the degradation rate of the EGFP mRNA, namely *d_m _*= 0.0173 min^-1 ^[7]).

Note also that the parameters found for Model 2 include a coefficient *h*_2 _= 0.126, hence less than unity. Since *h*_2 _describes the number of siRNA binding sites on the targeted mRNA, it should be greater than, or equal to, 1 in order to have a clear biological interpretation. However, if we constrain this parameter to be greater or equal to one, then the model optimizes at the value of *h*_2 _= 1, which makes Model 2 identical to Model 1.

**Figure 5:**
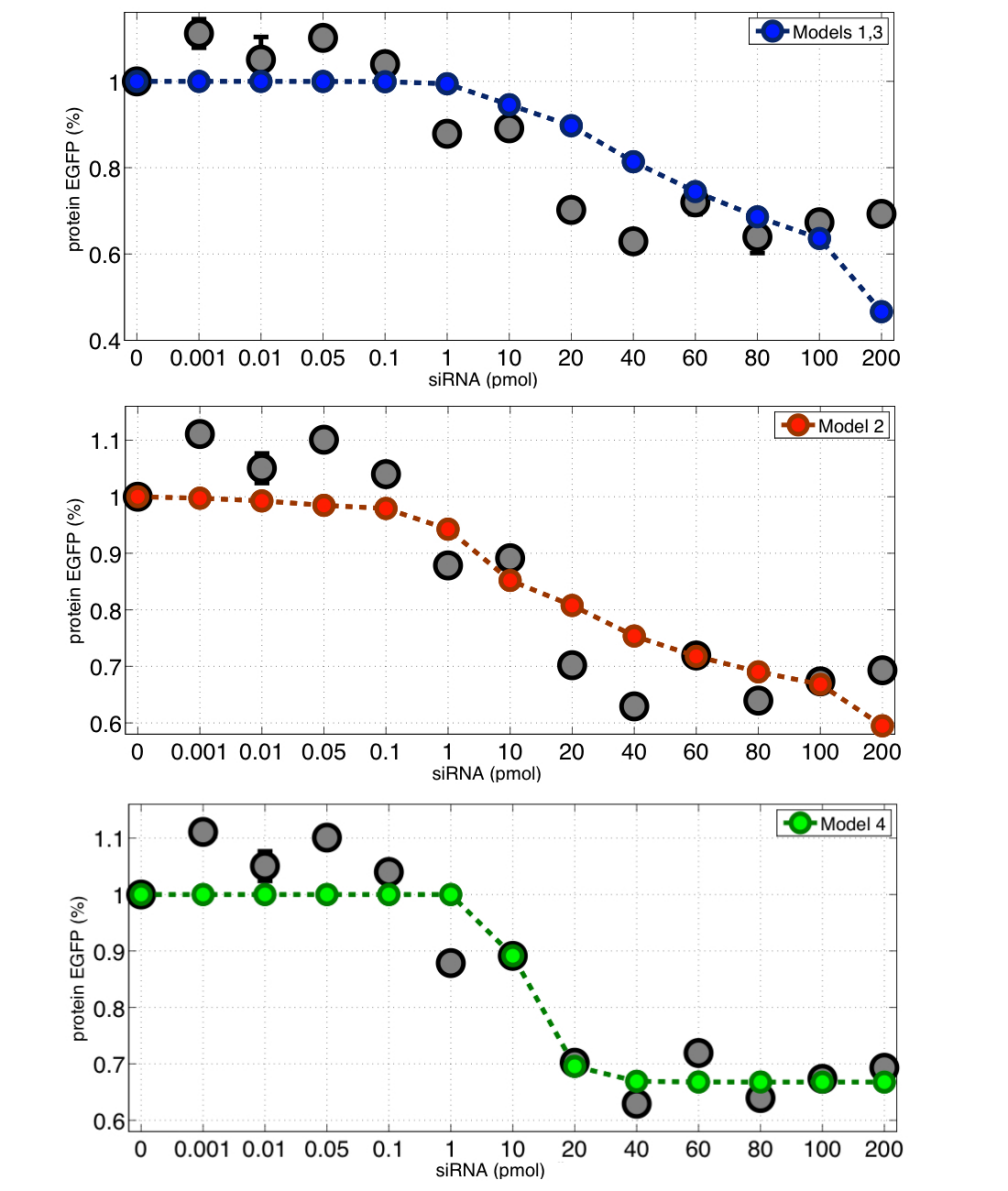
**Numerical fitting of the four models of the in vitro experimental results on protein EGFP levels presented on Figure 3**. The optimized parameter values and the corresponding fit error of each model are given in Table 2.

We have observed that in all numerical simulations, Models 1 and 3 are almost indistinguishable. The large optimized value of parameter c_3 _of Model 3 (namely *c*_3_/*k_m _*= 1.33 × 10^4^), the low value of parameter *k*_3_*h*_3 _= 1.40 × 10^-4 ^suggest *c*_3 _≫ *k*_3_*h*_3_*X_m_*, and hence the function *δ *(*X_s_, X_m_*) of Model 3 can be approximated by *k*_3_*h*_3_*X_s_X_m_*, which is nothing else than Model 1 (notice that the parameter optimization for Model 1 gives *k*_1 _≈ *k*_3_*h*_3 _in Table 2). Therefore, in this parameter space, Model 3 is almost identical to Model 1. Similar results were obtained when searching for the parameters which best fitted the measured protein levels (set II), shown in Figure 5. Model 4 is again the one with the smallest error. Model 1 and 3 are again unable to capture efficiently the experimental data. Model 2 is better than Models 1 and 3, but it is still worse than Model 4.

When we fitted the models to the third experimental dataset (set III), which was performed on a different cell-line, with both a different target mRNA and a different siRNA oligomer, Model 4 still performed better than the others with the smallest error, although Model 2 was a close match. Model 3 and 4 were again behaving very similarly and had the largest error. It should be noted that as it happened with the previous fitting results, also in this case Model 2 has a Hill coefficient smaller than unity (*h*_2 _= 0.126).

**Finally we observed, as shown in **Additional file 1Figure A1, that the experimental error for set III experiment was larger when compared to the *EGFP *experiment (set I). This was caused by the relative low expression of the *tTA *in the CHO cell lines, when compared to the *EGFP *expression in HEK cells, which made measurements more noisy.

### Assessing the model predictive ability

The models described above have the same number of unknown parameters to be learned (Methods), but for Model 4, which has one extra parameter. To be sure that the improved performance of Model 4 in describing the experimental data was not due to overfitting, we computed for each model and for each experimental dataset, the prediction error, which allows to assess the generalisation performance of the models [30]. We followed a "leave-one-out" cross validation strategy, where for each model and for each dataset, the parameter identification procedure was repeated each time removing one of the experimental points and then predicting the missing point with the identified parameters. We thus could estimate a prediction error for each model in each experimental dataset. This value is reported in Table 2and Additional file 1Table A1. Model 4 is again the one with the smallest prediction error, once again confirming this model superior performance in describing the data.

## Conclusions

Our findings show that the simple Hill function described by Model 4 is sufficient to quantitatively describe the effect of RNA interference, at the mRNA and protein level, in mammalian cells in vitro, for varying concentration of siRNA oligomers.

One significant feature of Model 4 is that it can predict the saturation effect of the RNAi process that we observed experimentally. We considered the possibility that this saturation could be in fact due to the inability of the cell to uptake high concentration of siRNA oligomers, however recent experiments [27], prove that uptake of siRNA oligomers in cells is linear with the concentration of siRNA oligomers transfected, at least in the concentration range we used. Additionally, Khan *et al *in [31], observed upregulation of mRNA targets of endogenous micro-RNA when transfecting siRNA oligomers in mammalian cells. In order to explain this effect, they suggested a saturation of the RISC complex (or other necessary small RNA processing or transport machinery).

It has been demonstrated in [23,26,27] that the enzymatic activity of RISC can be efficiently modeled in-vitro as a classic Michaelis-Menten reaction, where the target mRNA is the substrate, the siRNA-loaded RISC is the active enzyme (at a constant concentration), and the product is the degraded mRNA. This is one feature that Model 4 does not capture; namely for a fixed amount of siRNA-loaded RISC (i.e. *X_s_*), Eq.(11) should approximate the Michaelis-Menten in Eq.(10), instead Model 4 becomes simply proportional to *X_m_*, as Model 1 and 2. Nevertheless, Model 4 approximates very well the experimental data. We believe this happens because enzymatic reactions have a typical *K_M _*much greater that the physiological concentration of their substrate (*X_m_*) [23], and the same happens for the RISC complex [23,26]. In this condition, the Michaelis-Menten equation becomes $V_{m}\frac{X_{m}}{K_{M}+X_{m}}≊\frac{V_{M}}{K_{M}}X_{m}$ and therefore it is linear in *X_m_*, as predicted by Model 4. When the siRNA concentration varies, Model 4 predicts that the parameters $\frac{V_{M}}{K_{M}}$ will change as a function of *X_s _*as described by Eq.(11).

The three parameters of Model 4 have a straightforward biological interpretation, and their values can be easily tuned to accommodate for different efficiencies of RNAi. For example, the parameter *d*_4 _can be used to weigh the degradation due to the RNAi compared to the endogeneous mRNA degradation, and its strength, i.e. what is the maximal degradation rate that can be achieved. *θ*_4 _quantifies the siRNA oligomers concentration needed to achieve half of the maximal degradation of the targeted mRNA. The *h*_4 _coefficient can accommodate for multiple target sites on the same mRNA, or for the cooperativity of the RISC complex.

Clearly, the RNAi process is very complex and no one-to-one relationship can be found between parameters of Model 4 and RNAi biological components. Nevertheless, it has been shown in [27] that between 10^4 ^and 10^5 ^siRNA oligomers per cell (corresponding to a concentration in the range 10 pM-100 pM) are sufficient to reach half-maximal mRNA target degradation. Model 4 predicts that half-maximal degradation is achieved for an amount of siRNA oligomers equal to *θ*_4_. The value of this parameter when fitting mRNA levels (Table 2 and Additional file 1 Table A1) is *θ*_4 _≈ 0.1 pmol despite of the different cell-lines and mRNA-siRNA pairs tested (EGFP and tTA). This value corresponds to a concentration of 50 pM in our experimental setting, hence in good agreement with the previously reported range. Altogether these observations suggest that the quantity *θ*_4 _could be cell-type, mRNA, and siRNA indipendent.

It is estimated that the concentration of active RISC in a cell is about 3 - 5 nM [23,26,32]. Taking into account that the volume of a mammalian cell is in the range 10^-13^*L *- 10^-12^*L*, then we can estimate that the number of active RISC in a cell is in the range 10^3 ^- 10^4^. The above observations suggest that saturation begins when the number of siRNA oligomers in a cell becomes comparable to the number of RISC molecules.

We observed that the parameters of Model 4 estimated when fitting protein levels (set II experiments) are very close to the ones estimated when fitting mRNA levels (set I experiments). Namely, the optimized values of *d*_4 _and *h*_4 _are very similar for both experimental data. This is important since these are two independent biological experiments. This proves the mathematical robustness of Model 4. The only parameter changing between the two sets of experiment is *θ*_4_, which represents the concentration of siRNA oligomers needed to achieve half of the maximal degradation rate (*d*_4_). This is reflected in Figure 2and Figure 3, where it is clear that saturation is achieved at about 1 pmol for the mRNA data (Figure 2) and at about 20 pmol for the protein data (Figure 3). This difference may be due to biological variability, or to the simplified model of protein translation dynamics we used (steady-state approximation).

We also conformed that Model 4 is cell-line-independent, mRNA-independent, and siRNA-independent, since it can accurately describe the RNA interference process on a different cell-line (CHO) expressing a different mRNA (tTA), silenced by a different siRNA oligomer.

Interestingly, the difference in Model 4 parameters, when testing a different mRNA-siRNA pair (i.e. tTA versus EGFP), shows that only *d*_4 _(the maximal degradation rate) and *h*_4 _(the cooperativity) change significantly, suggesting that these two parameters can be used to describe changes in siRNA-mRNA silencing specific strength, whereas *θ*_4 _may be kept constant.

Recently it has been proposed that siRNA and microRNA efficacy, defined as the percentage decrease in the target mRNA level due to the silencing reaction, could be limited due to mRNA abundance [33]or to mRNA degradation rate [34].

Model 4 predicts that the percentage decrease in target mRNA level (obtained from Eq. (13) simply dividing by *k_m_*/*d_m_*) is indeed sensitive to *d_m _*(the target mRNA degradation rate), with a higher degradation rate corresponding to a weaker effect of the silencing reaction, and vice-versa. This result is in line with the experimental observation described in Larsson et al [34]. In addition, according to Model 4, the transcription rate *k_m _*of the target mRNA does not have any influence on the silencing reaction efficacy. The target mRNA abundance, in absence of the silencing reaction, is simply obtained from Eq. 1 as *k_m_*/*d_m_*. Smaller *d_m _*will correspond to a higher mRNA abundance (for a constant *k_m_*) therefore a correlation between mRNA abundance and sensitivity to mRNA can be found [33], but this is only an indirect effect mediated by the degradation rate, at least according to our model. Our conclusion is that siRNA-mediated degradation in mammalian cells can always be best represented as an enzymatic reaction described by an Hill function, whose parameters have to be tuned to the specific siRNA-mRNA pair.

The models discussed so far consider the average behavior of a population of cells. In the case of singe-cell experiments, these models might not be efficient enough due to their deterministic nature and will not be able to capture any stochastic effects.

Since RNA has a plethora of functional properties and plays many of roles in regulating gene expression, it has been used in a number of different studies as a tool for elucidating gene functions. In fact with RNAi it is possible to selectively knock-down any gene and even modulate its dosage [35]. RNA has also been used in the design of therapeutic molecules as well as metabolic reprogramming [36]. The potential uses of this versatile molecule are still very much under study, but their effectiveness depend on many variables such as, the concentration of the silencing reagent, the transfection techniques, the cell type used and the target type selection. In the present study we biologically validated for the first time a mathematical model (Model 4) that has a simple mathematical form, amenable to analytical investigations and a small set of parameters with an intuitive physical meaning that can be used both by the computational and the experimental community interested in the analysis and application of RNA interference.

## Methods

### RNA interference by small interfering oligonucleotides (siRNA)

The sequence of the 21-mer siRNA double-stranded oligomers targeting EGFP was identical to the one reported in [37]. This siRNA targets the coding sequence of the EGFP gene starting at position 237 from the ATG, on the target sequence AAGCAGCACGACTTCTTCAAG. The siRNA HPLC purified, with sequence GCAGCACGACUUCUUCAAGtt (concentration 100 *μ*M) was synthesized by Ambion. As a negative control we used, in all experiments a shuffled sequence non targeting siRNA from Dharmacon. A 21-mer siRNA oligonucleotide was designed against the coding sequence of tetracycline-controlled transactivator (tTA) gene using the Ambion technology platform. Custom siRNA HPLC purified with sequence GGUUUAACAACCCGUAAACtt (concentration 100 *μ*M) were synthesized by Ambion on the target sequence AAGGTTTAACAACCCGTAAAC starting at position 57 from the ATG in the tTA gene coding sequence.

### Cell culture and transfection

HEK 293 stably expressing EGFP (kindly provided by Mara Alfieri) were maintained at 37°C in a 5% CO2-humidified incubator. HEK 293 cells were cultured in Dulbecco's modified Eagle's medium (DMEM, GIBCO BRL) supplemented with 10% heat-inactivated fetal bovine serum (FBS, Invitrogen) and 1% antibiotic/antimycotic solution (GIBCO BRL). CHO AA8 Tet-Off Cell Line (Clontech) stably expressing the tetracycline-controlled transactivator (tTA) were maintained at 37degC in a 5% CO2-humidified incubator. CHO cells were cultured in alpha-MEM (GIBCO BRL) supplemented with 10% heat-inactivated fetal bovine serum (FBS) (Invitrogen) and 1% antibiotic/antimycotic solution (GIBCO BRL). Cells were seeded at a density of 300.000 per well in a 6 wells multi-well and transfected 1 day after seeding using Lipofectamine 2000 (Invitrogen) according to manufacturer's instructions with siRNA (Silencer Custom siRNA, 100 *μ*M, Ambion) in a range of quantities from 0.001 pmol to 200 pmol (total concentration). The amounts of transfected siRNA oligomers were: 0, 0.001, 0.01, 0.05, 0.1, 0.5, 1.0, 10.0, 20.0, 40.0, 60.0, 80.0, 100.0 and 200.0 pmol in a total of 2 mL of medium (so the final concentrations of siRNA oligomers were 5 × 10^-4^, 5 × 10^-3^, 2.5 × 10^-2^, 5 × 10^-2^, 2.5 × 10^-1^, 5 × 10^-1^, 5.0, 10.0, 20.0, 30.0, 40.0, 50.0, and 100 nM respectively). Each experiment was performed in biological triplicates, and the resulting standard deviations were computed and reported in each graph. One day post-transfection, the media and ligand were replaced. Transfected cells were collected 48 hours post-transfection for RNA extraction and subsequent analysis. FACS analysis was performed 60 hours after transfection.

### RNA extraction and Real-time PCR

Total RNA extraction from 35 mm culture plates was performed using the Qiagen RNeasy Kit (Qiagen) according to manufacturers instructions. Retro-transcription of 1 *μ*g of the total RNA extracted was performed using the QuantiTect^®^Reverse Transcription Kit (Qiagen), according to manufacturers instructions. Quantitative real-time PCR was performed using a LightCycler (Roche Molecular Biochemicals, Mannheim, Germany) to analyze the amplification status of EGFP and tTA. Amplification of the genes was performed from the cDNA obtained from the total RNA and using the LightCycler DNA Master SYRB Green I kit (Roche Molecular Biochemicals). Primer sequences for Human GAPDH and Chinese Hampster GAPDH (used as reference genes) were designed by Primer 3.0 http://frodo.wi.mit.edu/ (Forward primer Human GAPDH: GAAGGTGAAGGTCGGAGTC; Reverse primer Human GAPDH: GAAGATGGTGATGGGATTTC; Forward primer Hamster GAPDH : ACCCAGAAGACTGTGGATGG; Reverse primer Hamster GAPDH: GGATGCAGGGATGATGTTCT). Primer sequences for EGFP and tTA were also designed with Primer 3.0 (Forward primer EGFP: ACGACGGCAACTACAAGACC; Reverse primer EGFP: GCATCGACTTCAAGGAGGAC; Forward primer tTA: ACAGCGCATTAGAGCTGCTT; Reverse primer tTA: ACCTAGCTTCTGGGCGAGTT). The relative amounts of genes were compared with the GAPDH reference genes and calculated using the Principle of Relative Quantification Analysis according to the standard formula 2^-*DCt*^. To confirm the specificity of the amplification signal, we considered the primer dissociation curve in each case.

### FACS analysis

Cells from 35 mm culture plates were trypsinized, filtered and subjected to Fluorescence-Activated Cell Sorting (FACS) analysis 60 hours posttransfection in a Becton Dickinson FACSAria.

### Models

In the context of the specific in vitro experiments we carried out, we can make the following assumptions to derive the mathematical model:

1. Cells express the target mRNA at a constant rate *k_m _*which corresponds to the maximum transcription rate of the promoter.

2. We assume that the siRNA oligomers will be quickly loaded into the RISC and that step 2 of Figure 1 takes place in much shorter time scale than steps 3 and 4.

Therefore, the steps 2 - 4 of RNA interference mechanism as shown in Figure 1 can be described by Equations 1. The negative control experiments involved the addition of non-specific siRNA oligomers, which are not complementary to the target mRNA and therefore are not able to trigger the RNA interference mechanism. Namely, *δ *(*X_m_, X_s_*) = 0. Therefore, the equations corresponding to the negative control experiments are:

$$[\text{mRNA}]:\;\;\;\frac{dX_{m}}{dt}=k_{m}-d_{m}X_{m},$$

(12) $$\left[\text{protein}\right]:\;\;\;\frac{dX_{p}}{dt}=k_{T}X_{m}-d_{p}X_{p},$$

#### Steady-state equations

For the numerical fitting of the in vitro experiments we used the steady state equations for the mRNAs or proteins. For example, for the in vitro experiments on RNA levels, the experimental period of 48 hours before extracting the RNA is considered long enough for the mRNAs to approach their equilibrium value. In order to solve for the mRNA or protein steady state we assume that siRNA concentration remains constant through the 48 hours of the in vitro experiments. In general, the siRNA-RISC complex, is considered very stable and one can assume that the degradation of siRNA is so slow that it does not have any effect on the overall dynamics. The steady state equations for the mRNA concentrations of the four models are:

(13) $$\begin{array}{l} \text{Model}\;\;1:{\tilde{X}}_{m}\;=\frac{k_{m}}{d_{m}+k_{1}X_{s}}\\ \text{Model}\;\;2:{\tilde{X}}_{m}=\frac{k_{m}}{d_{m}+k_{2}^{h_{2}}X_{s}^{h_{2}}}\\ \text{Model}\;\;3:{\tilde{X}}_{m}\;=\frac{-B+\sqrt{B^{2}+4c_{3}k_{3}h_{3}k_{m}d_{m}}}{2k_{3}h_{3}d_{m}}\;\;\text{where}\;B=\left(c_{3}d_{m}+c_{3}k_{3}h_{3}X_{s}-k_{3}h_{3}k_{m}\right)\\ \text{Model}\;\;4:{\tilde{X}}_{m}\;=\frac{k_{m}(\theta^{h_{4}}+X_{s}^{h_{4}})}{\theta^{h_{4}}d_{m}+(d_{4}+d_{m})X_{s}^{h_{4}}} \end{array}$$

The corresponding mRNA equilibrium of the negative control experiments is simply *X_m _= k_m_/d_m _*(for all the models since *δ *(*X_m_, X_s_*) = 0). Therefore, when fitting the ratio of the mRNA levels between positive and negative control, we multiply equations (13) by *d_m_/k_m_*. For models 1,3 and 4 this results in the cancellation of term *k_m_*, making the numerical fitting independent of the strength of the promoter. However, this is not the case for Model 3 (because these terms cannot be cancelled out). Throughout the numerical optimization, the degradation rate of mRNA EGFP was fixed at the value of *d_m _*= 0.0173 min^-1^, which corresponds to a half-life of 40 minutes, as estimated in [7]. We used the same value of *d_m _*also when fitting the experimental datset III for the *tTA *mRNA since this is in the reported range for this mRNA as well [7]. In order to optimize Model 3 with the smallest possible number of parameters, we clustered its 4 different parameters (*k*_3_, *h*_3_, *c*_3_, *k_m_*) in order to have only two optimized quantities: *k*_3 _*h*_3 _and *c*_3_/*k_m_*. For the in vitro experiments in protein levels, we fitted numerically the protein steady-state equations. The equilibrium concentration of protein is given by:

(14) $$X_{p}=\frac{k_{T}}{d_{p}}{\tilde{X}}_{m}$$

where ${\tilde{X}}_{m}$ is the mRNA equilibrium, which is different for each model (equations 13). Additionally, the protein steady-state of the negative control model is:

(15) $$X_{p}=\frac{k_{T}}{d_{p}}\frac{k_{m}}{d_{m}}.$$

For the numerical fitting of the ratio of protein levels between negative and positive control, one needs to divide equation (14) by equation (15).

#### Parameter fitting and Prediction Error

For the numerical fitting of the mRNA levels from in vitro experiments, we used the following error function:

(16) $$\sum_{i}^{N}{\left(\frac{Y_{model}^{i}-Y_{data}^{i}}{SE^{i}}\right)}^{2}$$

where *N *is the number of experimental points, $Y_{data}^{i}$ is the experimental measurement of experiment *i*, $Y_{model}^{i}$ model is the model prediction for experiment *i *and SE*^i ^*is the standard error of experiment *i*. A genetic algorithm implemented in the "Genetic Algorithm and Direct Search Toolbox" of Matlab (the *ga *command) was then used to find the parameters which minimised the error function.

The absolute value errors of each model were then normalized against the largest error. Namely, the error of Model 3 (which in both case was the largest one) was set to 1 and all the other errors of the remaining three models, were normalized against error of Model 3.

The Prediction Error (PE) for each experimental dataset was computed by repeating the parameter fitting procedure described above, but this time using a leave-one-out cross-validation procedure. The PE was then computed as the average error (Eq. 16) between the predicted value and the experimental value across all the experimental points. As done for the error function, we then computed a relative value for the PE in order to compare the performance across the different models by normalising against the maximum PE across the four models, and reported it in Table 2 and in Additional file 1 Table A1.

Please observe that in the case of the *tTA *mRNA the cost function used was as in Eq. 16 but without dividing by the standard error *SE *since in this case the experimental data were more noisy and the *SE *could not be estimated accurately.

## Authors' contributions

GC and VS designed the experiments. GC, VS and MG performed the experiments. AP developed the models and AP and DdB conducted simulations. AP, GC and DdB drafted the manuscript. DdB and MdB conceived and supervised the collaboration and overall strategy of the project and edited the manuscript. All authors have read and approved the final manuscript.

## Supplementary Material

Additional file 1 Supplementary material for Modeling RNA interference in mammalian cells. Results and fitting of in vitro experiments on hamster ovary cell line (CHO) constitutively expressing tTA protein. We measured mRNA levels, by quantitative Real-Time PCR for a large range of concentrations of siRNA oligomers, from 0.001 pmol to 200 pmol (total concentration). The amounts of transfected siRNA oligomers were: 0, 0.001, 0.01, 0.05, 0.1, 0.5, 1.0, 10.0, 20.0, 40.0, 60.0, 80.0, 100.0 and 200.0 pmol in a total of 2 mL of medium (so the final concentrations of siRNA oligomers were 5 × 10^-4^, 5 × 10^-3^, 2.5 × 10^-2^, 5 × 10^-2^, 2.5 × 10^-1^, 5 × 10^-1^, 5.0, 10.0, 20.0, 30.0, 40.0, 50.0, and 100 nM respectively). Each experiment was performed in biological triplicates, and the resulting standard deviations are computed and reported in each graph. In Additional file 1 Table A1, numerical fitting results and predicted error for the four models, in Additional file 1 Figure A1, graphic representation of the numerical fitting. Click here for file
