# Supplementary material for: Modeling RNA interference in mammalian cells
Source: BMC Syst Biol. 2011 Jan 27;5:19. doi: 10.1186/1752-0509-5-19 (PMC3040133; doi:10.1186/1752-0509-5-19)
Supplement: Additional file 1 — Supplementary material for Modeling RNA interference in mammalian cells. Results and fitting of in vitro experiments on hamster ovary cell line (CHO) constitutively expressing tTA protein. We measured mRNA levels, by quantitative Real-Time PCR for a large range of concentrations of siRNA oligomers, from 0.001 pmol to 200 pmol (total concentration). The amounts of transfected siRNA oligomers were: 0, 0.001, 0.01, 0.05, 0.1, 0.5, 1.0, 10.0, 20.0, 40.0, 60.0, 80.0, 100.0 and 200.0 pmol in a total of 2 mL of medium (so the final concentrations of siRNA oligomers were 5 × 10^-4^, 5 × 10^-3^, 2.5 × 10^-2^, 5 × 10^-2^, 2.5 × 10^-1^, 5 × 10^-1^, 5.0, 10.0, 20.0, 30.0, 40.0, 50.0, and 100 nM respectively). Each experiment was performed in biological triplicates, and the resulting standard deviations are computed and reported in each graph. In Additional file 1 Table A1, numerical fitting results and predicted error for the four models, in Additional file 1 Figure A1, graphic representation of the numerical fitting. [file 1752-0509-5-19-S1.PDF]

Supplementary Material for Modeling RNA interference in mammalian cells

Giulia Cuccato[1,♯], Athanasios Polynikis[2,♯] , Velia Siciliano[1], Alda Graziano[1], Mario di Bernardo[2,3] and Diego di Bernardo[*1,3]

[1]Telethon Institute of Genetics and Medicine (TIGEM), Naples, Italy
[2]Department of Engineering Mathematics, University of Bristol, Bristol, United Kingdom
[3]Department of Computer and Systems Engineering, University of Naples Federico II, Naples, Italy
♯ These authors contributed equally to this work.

Email: Giulia Cuccato - cuccato@tigem.it; Athanasios Polynikis - Th.Polynikis@bristol.ac.uk; Velia Siciliano - siciliano@tigem.it; Alda Graziano - graziano@tigem.it; Mario di Bernardo - mario.dibernardo@unina.it; Diego di Bernardo - dibernardo@tigem.it;

[*]Corresponding author

Table 1: Numerical fitting results of the four models for in vitro experimental data. The relative error for each model is given, together with the corresponding optimized values of its parameters. The unit of measurements are reported for the dimensional parameters . (a.u. stands for arbitrary units of concentration)

**Experiment on *tTA* mRNA levels**					
	**Fit Err.**	**Pred. Err.**	**Parameters**		
**Model 1**	0.92	0.95	$k_1 = 2.42 \times 10^{-4}(\text{a.u.}\cdot\text{min})^{-1}$,		
**Model 2**	0.15	0.19	$k_2 = 1.00 \times 10^{-2}(\text{a.u.}\cdot\text{min})^{-1}$,	$h_2 = 0.113$,	
**Model 3**	1	1	$k_3h_3 = 4.61 \times 10^{-4}(\text{a.u.}\cdot\text{min})^{-1}$,	$c_3 = 1.36 \times 10^3\text{min}^{-1}$,	
**Model 4**	0.10	0.18	$\theta_4 = 0.051\text{pmol}$,	$d_4 = 0.016\text{min}^{-1}$,	$h_4 = 1.09$

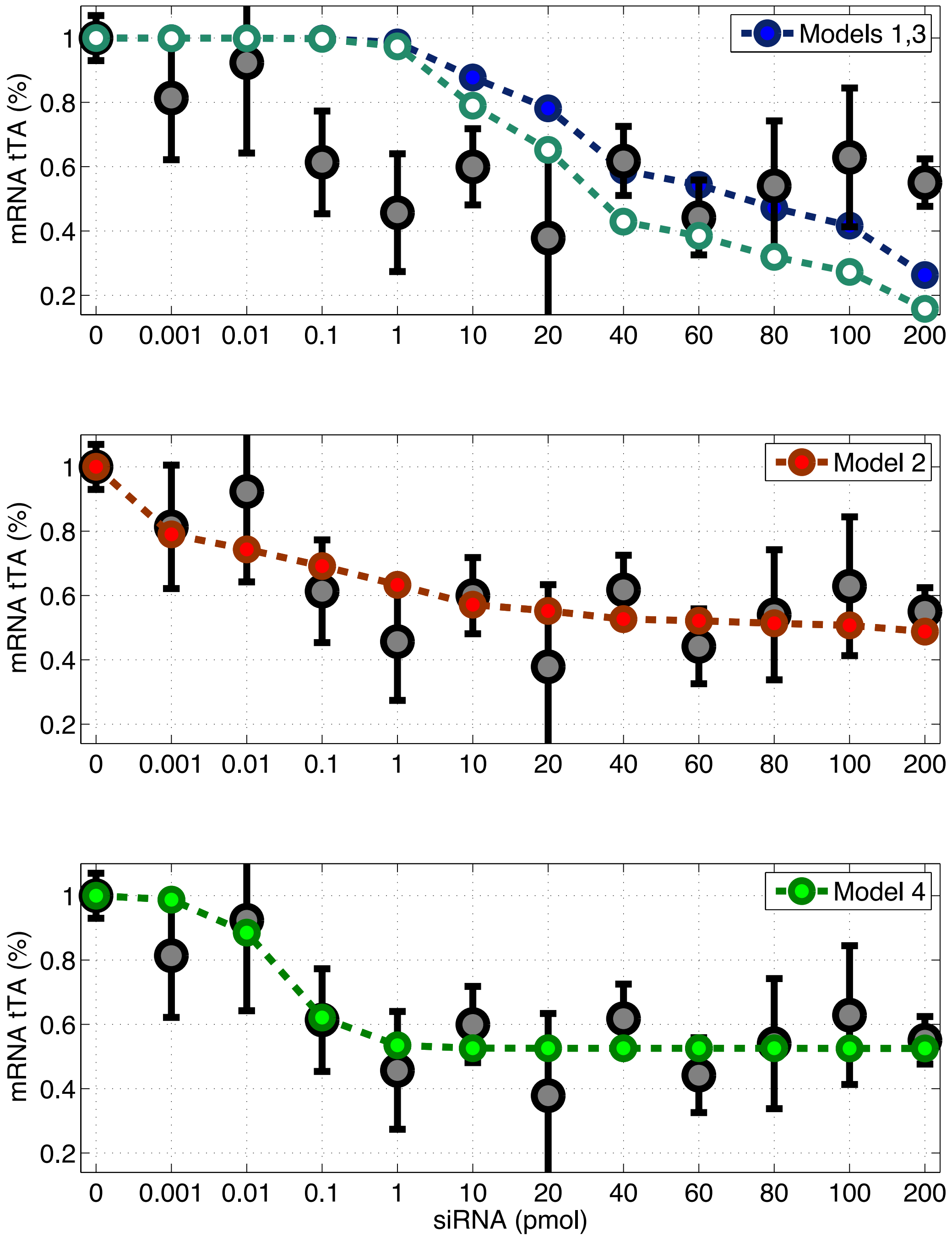

Figure 1: Numerical fitting of the four models on the in vitro experimental results on mRNA *tTA* expression levels (gray points with error bars). The optimized parameter values and the corresponding fit error of each model are given in Table 1
